# Navigating PROTACs in Cancer Therapy: Advancements, Challenges, and Future Horizons

**DOI:** 10.1002/fsn3.70011

**Published:** 2025-02-01

**Authors:** Saooda Ibrahim, Muhammad Umer Khan, Iqra Khurram, Raima Rehman, Abdur Rauf, Zubair Ahmad, Abdullah S. M. Aljohani, Waleed Al Abdulmonem, Mohammed Mansour Quradha

**Affiliations:** ^1^ Institute of Molecular Biology and Biotechnology The University of Lahore Lahore Pakistan; ^2^ Centre for Applied Molecular Biology University of the Punjab Lahore Pakistan; ^3^ Department of Chemistry University of Swabi Swabi Khyber Pakhtunkhwa Pakistan; ^4^ Department of Medical Biosciences College of Veterinary Medicine, Qassim University Buraydah Saudi Arabia; ^5^ Department of Pathology College of Medicine, Qassim University Buraydah Saudi Arabia; ^6^ College of Education, Seiyun University Seiyun Hadhramawt Yemen

**Keywords:** cancer, cancer therapy, oncogenic proteins, PROTACs

## Abstract

Proteolysis Targeting Chimeras (PROTACs) have revolutionized cancer therapy by offering a selective and innovative approach to degrade key oncogenic proteins associated with various malignancies. These hybrid molecules exploit the ubiquitin‐proteasome system, facilitating the degradation of target proteins through an event‐driven mechanism, thereby overcoming drug resistance and enhancing selectivity. With diverse targets including androgen receptors, BTK, estrogen receptors, BET proteins, and BRAF, PROTACs offer a versatile strategy for personalized cancer treatment. Advantages of PROTACs over traditional small molecule inhibitors include their ability to operate at lower concentrations, catalyzing the degradation of multiple proteins of interest with reduced cytotoxicity. Notably, PROTACs address challenges associated with traditionally “undruggable” targets, expanding the therapeutic landscape of cancer therapy. Ongoing preclinical and clinical studies highlight the transformative potential of PROTACs, with promising results in prostate, breast, lung, melanoma, and colorectal cancers. Despite their potential, challenges persist in optimizing physicochemical properties and enhancing bioavailability. Further research is needed to refine PROTAC design and address complexities in molecule development. Nevertheless, the development of oral androgen receptor PROTACs represents a significant milestone, demonstrating the feasibility and efficacy of this innovative therapeutic approach. This review provides a comprehensive overview of PROTACs in cancer therapy, emphasizing their mechanism of action, advantages, and challenges. As PROTAC research progresses, continued exploration in both preclinical and clinical settings will be crucial to unlocking their full therapeutic potential and shaping the future of personalized cancer treatment.

## Graphical abstract

Illustrating the mechanism of Proteolysis Targeting Chimeras (PROTACs), development of ligands available for PROTACs, PROTACs targeting cancers, and its advantages. PROTACs offer a versatile strategy for personalized cancer treatment

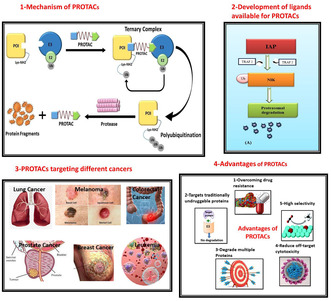

## 1 Introduction

Cancer is a group of diseases that originate from genetic and epigenetic abnormalities, causing uncontrolled cell growth and spread. In the United States, it ranks second in terms of mortality, with lung, prostate, breast, and colorectal cancers being the most prevalent (Siegel et al. 2021). According to the World Health Organization (WHO), approximately 10 million fatalities, accounting for one‐sixth of all deaths, were reported in 2020. Furthermore, roughly 400,000 children were diagnosed with cancer annually. In 2020, breast, lung, prostate, non‐melanoma skin, and stomach cancer were the most prevalent forms of the disease. The leading causes of cancer‐related deaths in 2020 included lung, colon and rectum, liver, stomach, and breast cancer (WHO 2023). Predictions for 2040 estimate that global new cancer cases will reach 30.2 million (WHO 2023). Conventional cancer treatments typically involve the use of anticancer drugs and surgery, but these methods often result in undesirable off‐target effects (Choudhary et al. 2023; Khurram et al. 2025).

Cancer pharmacotherapy typically encompasses (i) targeting rapidly dividing cells by inducing DNA damage, replication stress, or disrupting the cytoskeleton; (ii) blocking sex hormone receptors or depleting their activating ligands; and (iii) molecularly targeted therapies that impede tumor growth, blood supply, or interfere with apoptosis and immune evasion (Johnston and Cheung 2018; Crawford et al. 2019; Buzun et al. 2020; Zhong et al. 2021). In recent years, the majority of newly approved cancer therapies have fallen within the molecularly targeted category, contributing to the avoidance of approximately 4.5 million cancer deaths between 1991 and 2019. Targeted cancer therapies concentrate on biomolecules that are unique to cancer, such as oncoproteins, with the aim of obstructing their harmful activities in cancerous tissues. Over the past few decades, these therapies have made remarkable progress and have become a powerful treatment option for cancer patients. Examples of these therapies include small molecular inhibitors and monoclonal antibodies that effectively target over expressed or overactive proteins found in cancer. Although these therapies have shown promising results, there are still challenges that need to be addressed. These challenges include limited therapeutic benefits, drug resistance, and off‐target effects. Despite these challenges, researchers are actively exploring new and more effective methods for targeting cancer‐related biomolecules (Gharwan and Groninger 2016; Khurram et al. 2024).

Protein homeostasis is a critical aspect of normal cellular function and relies on the precise regulation of protein activity, localization, and abundance (Ballabio and Bonifacino 2020; Balch et al. 2008). Protein ubiquitination plays a central role in these processes, as the ubiquitin proteasome system employs various proteins to polyubiquitinate substrates for targeted proteolytic degradation via the 26S proteasome, thereby influencing protein abundance and function (Kleiger and Mayor 2014). In cancer research, understanding proteolytic regulation mechanisms is essential, as efforts are focused on utilizing the ubiquitin proteasome system to selectively target and degrade proteins, especially oncoproteins, for therapeutic purposes. Among the various proteolytic approaches currently under development, PROTACs (proteolysis targeting chimeras) are one of the most advanced methods to date (Burke, Smith, and Zheng 2022; Békés, Langley, and Crews 2022; Zhao et al. 2022).

PROTACs, often referred to as hybrid chimeric molecules, have emerged as a noteworthy approach in the field of cancer treatment due to their innovative strategy for selectively degrading proteins (Choudhary et al. 2023). These bifunctional degrader molecules, known as proteolysis targeting chimeras (PROTACs), function by bringing a target protein into proximity with a recruited E3 ubiquitin ligase. This proximity ultimately results in the ubiquitination and subsequent degradation of the target protein (Meyers et al. 2017). PROTACs operate by directly targeting cellular proteins for degradation through interaction with the E3 complex. These heterobifunctional molecules are composed of three components: a ligand for binding to POIs, a ligand for recruiting the E3 complex, and a linker connecting both. During action, PROTACs facilitate the transfer of ubiquitins onto lysine residues of proteins of interest (POIs), leading to in situ poly‐ubiquitination (Sakamoto et al. 2001). The poly‐ubiquitinated POIs are then recognized and directed to proteasomes, where the ubiquitin chains are removed, and the POIs undergo degradation (Cromm and Crews 2017; Burslem and Crews 2017).

PROTACs exhibit advantages over traditional small molecule inhibitors (SMIs) in several aspects. One significant distinction is their unique mode of action, which is event‐driven pharmacology. PROTAC molecules can catalyze the degradation of numerous proteins of interest (POIs), operating at a lower concentration than SMIs for the desired pharmacological effect. This reduced concentration may mitigate the toxicities associated with SMIs. Moreover, PROTACs can target proteins that have historically been challenging to target, such as transcription factors (TFs). For instance, PROTACs designed for STAT3, an often undruggable TF, have recently been reported in the literature (Bai et al. 2019; Zhou et al. 2019).

PROTACs have shown effectiveness in overcoming drug resistance resulting from mutations in POIs. Examples of such mutations include those in proteins such as BCR‐ABL (Zhao et al. 2019), receptor tyrosine kinases (RTKs) (Burslem et al. 2018), estrogen receptor alpha (ERα) (Gonzalez et al. 2020), and Bruton's tyrosine kinase (BTK) (Buhimschi et al. 2018). Furthermore, PROTACs can address resistance to SMIs caused by upregulation of the target protein by degrading the protein itself. For instance, androgen receptor (AR) degraders have demonstrated potential in overcoming resistance to the AR antagonist enzalutamide during prostate cancer treatment (Kregel et al. 2020).

PROTACs have shown promising results in targeting various proteins associated with several diseases, such as cancer, viral infections, immune disorders, and neurodegenerative conditions. Notable examples include AstraZeneca's PROTACs targeting B‐cell lymphoma 6 (BCL6), GlaxoSmithKline's (GSK) efforts against P300/CBP‐associated factor and general control nonderepressible 5 (PCAF/GCN5), Pfizer's work on Bruton's tyrosine kinase (BTK), Boehringer Ingelheim's focus on focal adhesion kinase (FAK), and GSK's exploration of Interleukin‐1 receptor‐associated kinase 4 (IRAK4) (McCoull et al. 2018; Nunes et al. 2019).

A new method, which utilizes folate‐PROTACs, has been proposed in recent research. This technique involves coupling a folate group to Von Hippel–Lindau (VHL) ligands, which enables cancer cell‐specific delivery and targeted degradation of POIs within cancer cells. By preserving normal cells, this innovative approach paves the way for selective POI degradation in cancer therapy. Examples of folate‐PROTACs, such as folate‐ARV‐771, folate‐MS432, and folate‐MS99, illustrate the folate receptor‐dependent degradation of BRDs, MEKs, and ALK, respectively, within cancer cells. This strategy presents a promising avenue for selective POI degradation in cancer therapy (Liu, Chen, Liu, et al. 2021).

The field of PROTAC research is still in its early stages but has made remarkable strides in recent years. Since the initial report of the first PROTAC in 2001, the domain of targeted protein degradation through small molecules has advanced at a rapid pace, with a key focus on the development of PROTAC technology. Despite this progress, assessments of PROTACs as potential cancer therapies in both preclinical and clinical settings have been somewhat restricted until recently (Sakamoto et al. 2001).

This review aims to provide a comprehensive understanding of PROTACs' mechanism of action, their advantages over conventional small molecule inhibitors, and the challenges associated with their development. The diverse range of targets, including androgen receptors, BTK, estrogen receptors, BET proteins, and BRAF, underscores the versatility of PROTACs in tailoring therapeutic strategies for specific cancer types. Ultimately, the goal is to inspire further research and innovation, paving the way for personalized and effective cancer treatments.

## 2 Review Methodology

The methodology for this review involves a systematic examination of the current literature on Proteolysis Targeting Chimeras (PROTACs) in cancer therapy. A comprehensive search was conducted across major scientific databases, including PubMed, Scopus, and Web of Science, to identify relevant articles, reviews, and clinical studies published up to the knowledge cutoff date in 2024. The search strategy was defined using specific inclusion and exclusion criteria. Search terms included “PROTACs,” “cancer therapy,” “oncogenic proteins,” and related keywords were used to refine the search. Studies were included if they: (i) discussed the mechanism of action, design principles, or therapeutic application of PROTACs; (ii) focused on cancer treatment or oncogenic protein targeting; (iii) presented preclinical or clinical results related to PROTAC efficacy or challenges; and (iv) were published in English. Exclusion criteria included studies that: (i) focused solely on non‐cancer applications of PROTACs and (ii) provided insufficient data on their mechanism, challenges, or clinical outcomes. Articles identified through the database search underwent a two‐step screening process. First, titles and abstracts were reviewed for relevance to the topic. Second, full‐text articles were examined to confirm the inclusion of recent advancements in PROTAC research. Data extraction focused on studies elucidating PROTAC mechanisms, therapeutic advantages, potential challenges, and clinical outcomes to ensure a balanced and comprehensive understanding of the field.

## 3 Protein Degradation and Ubiquitin‐Proteasome System (UPS)

Protein degradation is integral in maintaining quality control as the proteins fold in a normal process within the cell. Most proteins are degraded in the ubiquitin‐proteasome system (UPS) (Nandi et al. 2006), of which they are tagged ubiquitin (Vijay‐Kumar, Bugg, and Cook 1987), a high conserved protein composed of 76 amino acids of 8.6 kDa, and directed by the 26S proteasome for degradation (Livneh et al. 2016). A series of three enzymes enable ubiquitination, a post‐translational modification (PTM).

In the first step, the E1 ubiquitin‐activating enzyme hydrolyses ATP to form an activated ubiquitin‐adenylate. It then proceeds to convert it into a thioester intermediate by reacting with a catalytic cysteine residue in the E1 enzyme. In the second step, the E2 enzyme transfers the ubiquitin to its catalytic cysteine via a transthioesterification reaction. Finally, in the third step, the ubiquitin is actually conjugated to the substrate protein. A ternary complex consisting of E2, E3 ubiquitin ligase and a target would deliver this ubiquitin to substrate protein. This leads to the isopeptide bond formation between the carboxy terminus of ubiquitin and the lysine residue on the target protein. In a class of E3 ligase, the ubiquitin can either be directly transferred from E2 to the substrate or can be sequentially relayed through E3 before finally reaching the substrate. In such a process, a series of ubiquitination may lead to the formation of a polyubiquitin chain on the substrate that gets recognized and degraded by the 26S proteasome.

PROTACs leverage the UPS to degrade a targeted protein. It has two ligands: one binding to the POI, and the other that recruits an E3 ligase, all of which are cross‐linked together to enable the ubiquitination and subsequent degradation of the POI (Li and Crews 2022).

## 4 Mode of Action of PROTACs

**FIGURE 1:**
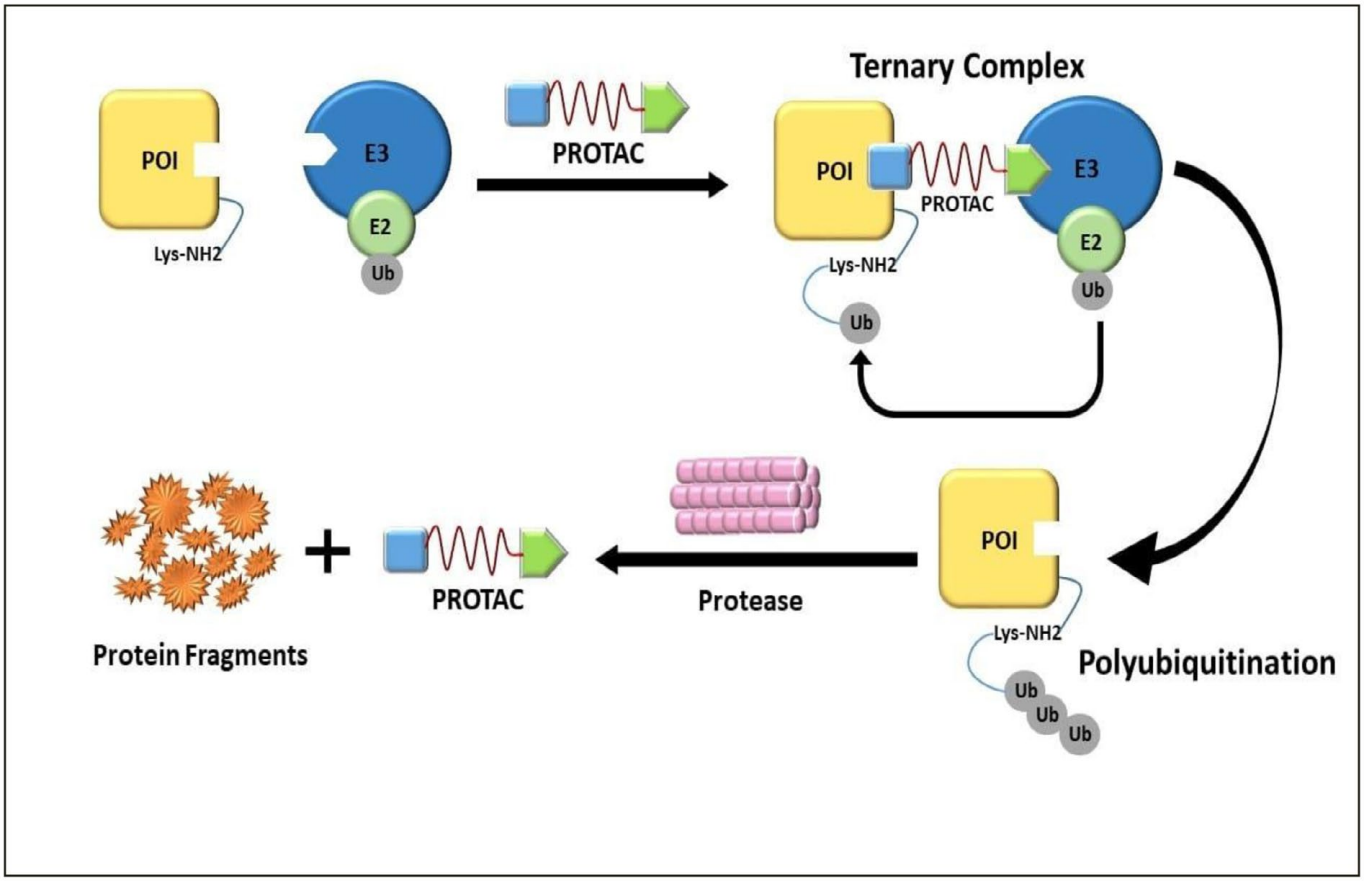
Mode of action of PROTACS.

While conventional molecular inhibitors operate on occupancy dependent manner by binding to the active site of targeted protein, PROTACs work on event‐based, sub‐stoichiometric mode of action (Zeng et al. 2021) as shown in Figure 1. PROTACs bind transiently to ubiquitin E3 ligase and the protein of interest (POI) and. After the degradation of POI, PROTAC detach from the complex and re‐enter the POI degradation cycle (Salami and Crews 2017). PROTACs are hetero‐bifunctional molecules composed of two parts: an “anchor” ligand that binds to an E3 ubiquitin ligase, and a “warhead” ligand that binds to a specific POI. The two ligands are connected by a chemical linker (Paiva and Crews 2019). The anchor ligand attaches to the substrate binding domain of the E3 ubiquitin ligase, while the warhead ligand binds to the target protein. This allows the E3 ubiquitin ligase to label and eliminate the target protein by ubiquitination, resulting in a decrease in its levels and activity (Paiva and Crews 2019).

PROTACs enter cell protoplasm and bind to POI and E3 ligase at their respective ligands, to form a E3 ligase‐PROTAC‐POI complex. This complex leads to the ubiquitination of the POI (Moreau et al. 2020). PROTACs function as intermediaries between E3 ligases and the targeted protein of interest (POI), by utilizing the natural protein degradation mechanism of the cell called ubiquitin‐proteasome system (UPS). Many E3 ligases are multi‐subunit complexes, typically comprising a catalytic subunit (Sc) and a substrate‐binding domain (SBD), which are connected by an adaptor (Ad) (Zhao et al. 2022).

Ubiquitination is a sequential reaction that is composed of two stages: polyubiquitination of POI and proteolysis of the complex by 26 proteosome enzymes (Nandi et al. 2006). It occurs when the ubiquitin‐activating enzyme E1 forms a high‐energy sulfur‐lipid bond with the ubiquitin by using ATP (Kwon and Ciechanover 2017). The activated ubiquitin is subsequently transferred to the ubiquitin‐conjugating E2 enzyme and is then, in the presence of E3 ubiquitin‐protein ligase, transferred to the POI by the formation of iso‐peptide bond of ubiquitin with the POI Lys residue (Guedeney et al. 2023). This leads to polyubiquitination, which is recognized by the 26S proteasome and transported to the 20S core (Myeku et al. 2016). The ubiquitin molecule hydrolyzes the target protein into oligopeptides and is released from the proteasome for degradation. The ubiquitin molecule then detaches from the substrates and returns to the cytoplasm for reutilization (Qi et al. 2021). By forming a binding affinity with both proteins in the cell, the PROTAC creates a ternary complex (TC) involving the E3 ligase (Ou et al. 2018). Initiating and maintaining the ubiquitination process depends on this ternary complex, which enables PROTACs to function catalytically—that is, one PROTAC molecule can destroy several targets (Moreau and Coen 2020). Unlike conventional drug activity that requires high dosage administration to exert clinically significant pharmacological activity, the sub‐stoichiometric mode of action allows PROTACs to eliminate the need for administering high drug dosage (Graham 2022). Furthermore, the rate of POI resynthesis and the stability of the PROTAC within the cell will determine if the mode of action of PROTACs provides a prolonged duration of action (Moreau and Coen 2020).

## 5 Design and Development of PROTACs

The degradation activity of PROTACs is determined by the binding affinity of both domains to their respective targets and the formation of stable E3 ligase‐PROTAC‐POI ternary complex (Liu, Hu, et al. 2022). However, a characteristic feature of PROTACs is that the binding site of the warhead on PROTACs is not critical for effective ubiquitination as long as it has sufficient affinity to attract the target protein to the ternary complex (Crews 2010). This feature allows PROTACs to target proteins previously thought of as undruggable (Xie et al. 2023). To date, PROTAC research has primarily focused on developing efficacious E3 binding ligands and applying the modality to novel POI targets (Zhao and Dekker 2022). Development of PROTAC has largely depended on empirical analyses and structure–activity relationships (SAR) studies. Xu et al. 2022 reported MS142, a VHL‐recruiting PROTAC AKT degrader, and MS5033, a novel CRBN‐recruiting PROTAC, as potent and selective degraders with high in vivo efficacy using SAR studies. These compounds exhibited rapid and robust AKT degradation via the ubiquitin‐proteasome system in a concentration‐ and time‐dependent manner, with cell proliferation inhibition and increased plasma exposure levels with no toxicity (Yu et al. 2022).

**FIGURE 2:**
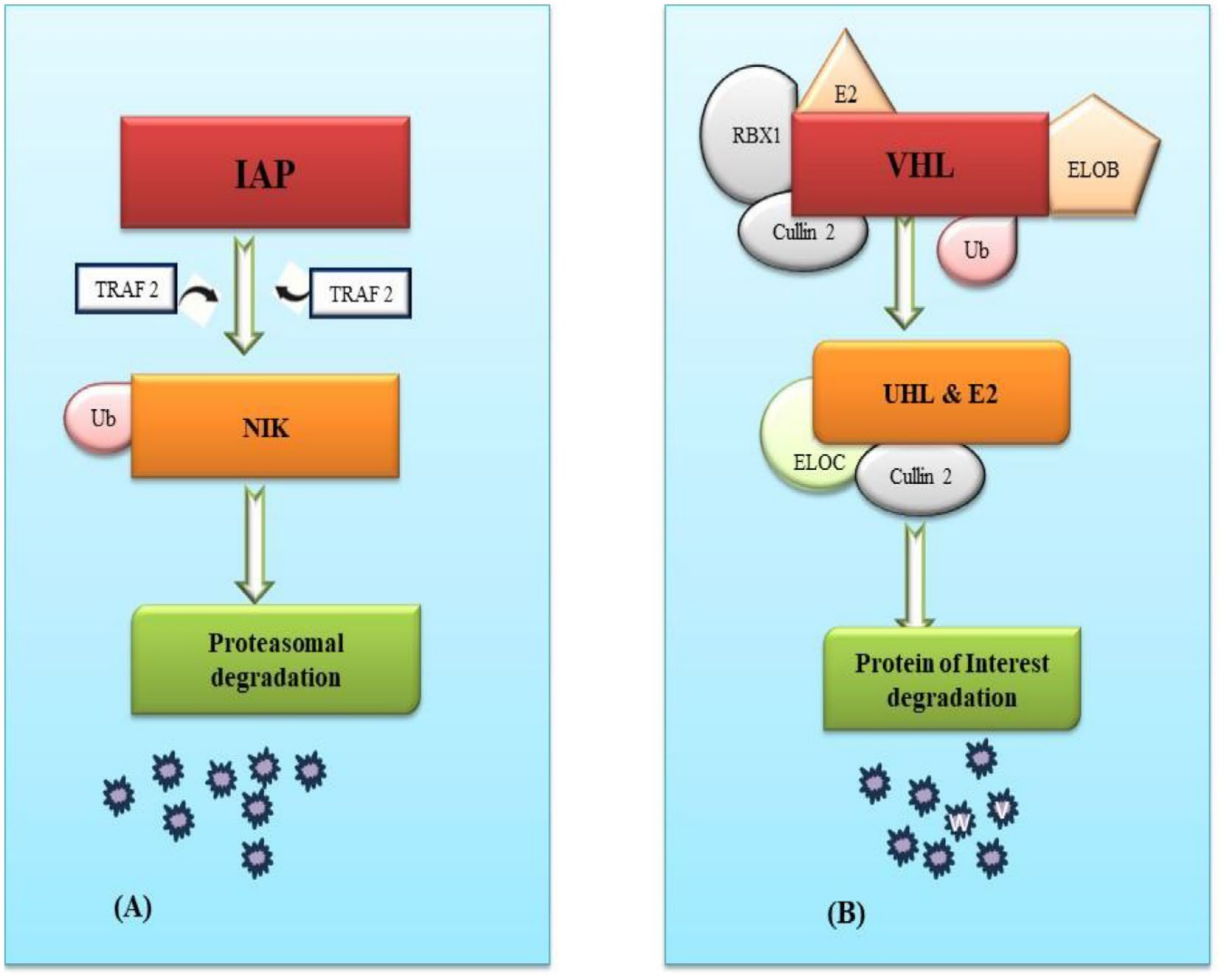
Design and development of ligands available for PROTACS.

**FIGURE 3:**
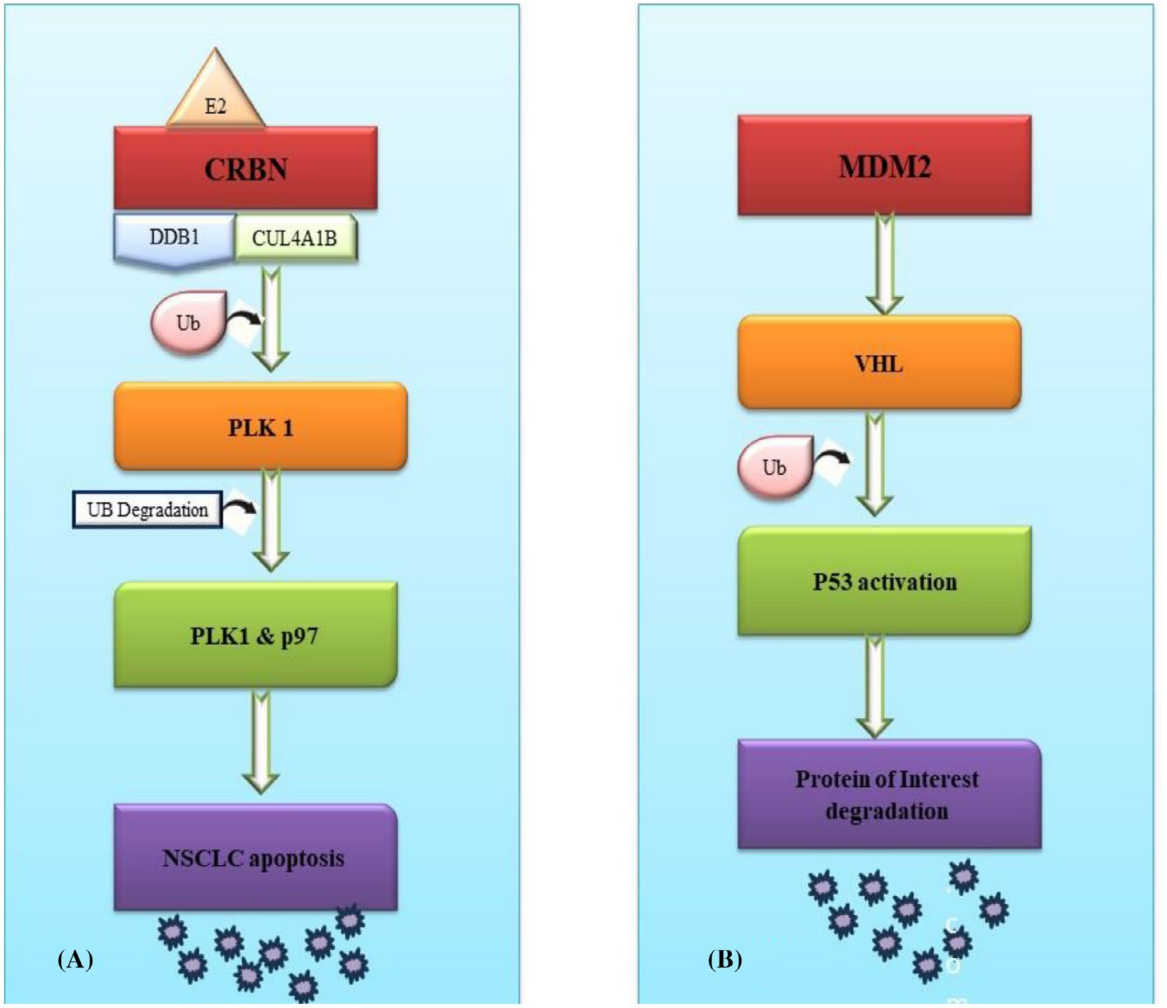
Design and development of ligands available for PROTACS.

Different recruited E3 ligases result in diverse degrees of protein degradation, primarily due to the distinct E3 ligases expression levels in different cells leading to dissimilar degradation efficiency. Additionally, some proteins have varying degrees of specificity for different E3 ligases (Bricelj et al. 2021). Although over 600 ligands of E3 ligase exist, only a limited number can be utilized with PROTAC technology to degrade POI, including SCFβ−TrCP, MDM2 (Murine double minute 2), IAPs (inhibitor of apoptosis proteins), VHL (Von Hippel–Lindau), and CRBN (cereblon) (Zhao et al. 2019). Comprehensive review of the PROTAC structures has reported that CRBN accounts for 60.1%, VHL for 30.1%, and cIAP (cyclic IAP) for 5.5% of the frequently used ligands [Cao]. CRBN‐targeted PROTACs are majorly preferred due to their high bioavailability, superior degradation efficiency, and more effective drug‐like properties, such as low molecular weight and high tissue permeability, as compared to other ligases. As a result, when designing PROTACs, ligands targeting CRBN or VHL are preferred, due to their diverse and extensive applications (Liu, Hu, et al. 2022) (Figures 2 and 3).

The improvement of degraders through the replacement of the warhead ligand has facilitated the rapid proliferation of the method for targeting proteins associated with a diverse array of diseases.

The development of degraders through the substitution of the warhead ligand has facilitated the rapid expansion of the methodology for targeting proteins associated with a wide range of diseases. More than 40 different cellular protein targets have now been reported to be targeted by degraders (Sun, Gao, et al. 2019). PROTAC degraders have been implicated in various cancers (Choudhary et al. 2023), autoimmune (Huang et al. 2023) and Alzheimer's diseases, a neurodegenerative disorder (George et al. 2023; Safdar et al. 2024). Furthermore, PROTACs POI have recently been extended beyond antivirals (Ahmad et al. 2023), antibiotics (Troup, Fallan, and Baud 2020) to agricultural applications (Leon and Bassham 2023). Advancement in PROTAC technology has resulted in the clinical trials of two degraders, ARV‐110 in metastatic castration‐resistant prostate cancer patients and ARC‐471 in ER+/ HER2‐locally advanced breast cancer patients (Liu et al. 2020).

It is now widely recognized that the potency of the degradation process is not solely dependent on the affinity of the warhead/anchor for E3 ligase and POI, but rather it is a consequence of the judicious pairing of the anchor and warhead through a suitable linker that promotes efficient TC formation and POI ubiquitination.

It is now widely recognized that the effectiveness of degradation is not solely dependent on the affinity of the warhead/anchor for E3 ligase and POI, but rather it results from the careful combination of the anchor and warhead through a suitable linker that promotes efficient TC formation and POI ubiquitination.

It is now widely recognized that the efficacy of degradation is not solely dependent upon the affinity of the warhead/anchor for E3 ligase and POI, but rather is a result of the careful pairing of the anchor and warhead through a suitable linker that promotes efficient TC formation and POI ubiquitination (Sun, Gao, et al. 2019; Burslem and Crews 2017). The linker, in terms of its length and composition, holds significant influence over the efficacy of TC formation, degradation activity, and target selectivity. Furthermore, the linker can also contribute to the cooperative binding of the POI, particularly for PROTACs with low affinity warheads (Roy et al. 2019). Currently, there is no established protocol for de novo design of PROTAC linker that warranties the generation of a highly effective degrader for a specific E3‐POI pairing, and a measure of empirical experimentation is often necessary. Despite this, the majority of PROTAC linkers historically have been constructed from only a small number of fundamental chemical structures (Troup, Fallan, and Baud 2020).

## 6 Therapeutic Role of PROTACS in Cancer

Maintaining proliferative signals, avoiding growth inhibitors, preventing cell death, initiating angiogenesis, and triggering metastasis and invasion are all hallmarks of the complicated process that leads to the development and spread of cancer (Hanahan and Weinberg 2011; Khurram et al. 2023; Saleem et al. 2024). Some overexpressed or over‐activated proteins have indeed been demonstrated to be important in the development of carcinogenesis and serve as prospective therapeutic targets. PROTACs, which target proteolysis, is a tool for carrying out treatment strategies. It eliminates or lessens the presence of pathogenic proteins and evolved as the potential therapeutic strategy for malignancies (Memon and Patel 2022).

## 7 PROTACS Targeting Different Proteins in Cancer

**FIGURE 4:**
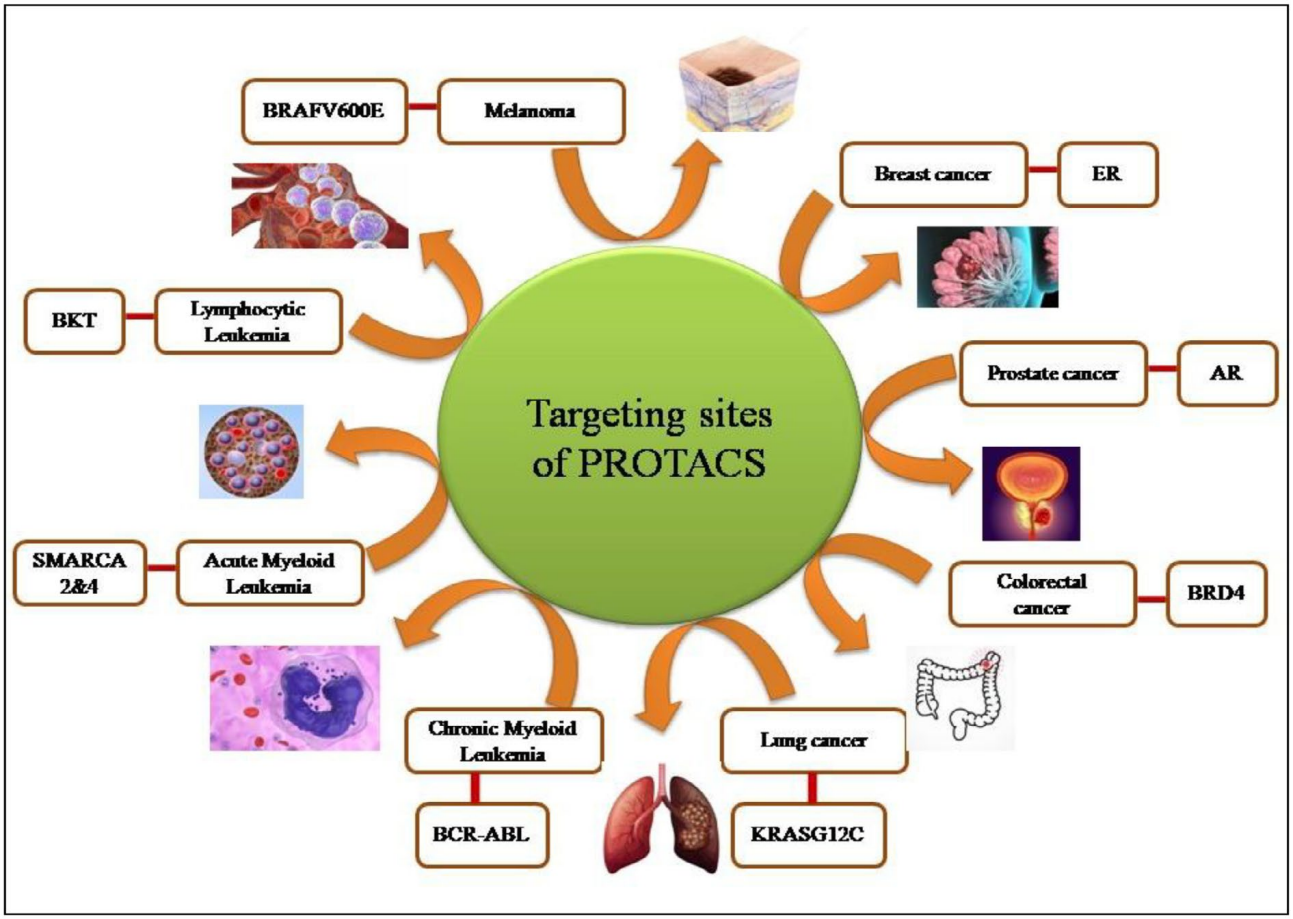
Targeting sites of PROTACS in various cancers.

With a focus on the proteins for treating cancer, more than 30 proteins crucial for the growth of malignancies have been identified yet (Ottis and Crews 2017; Itoh 2018; Gu et al. 2018). Protacs targeted proteins include; I‐nuclear receptors (such as AR and ER), II‐proteins involved in regulations (e.g., AHR, X‐protein, ERRα, CRABP‐I, II, and TACC3), III‐ a protein involved in the regulation of transcription (such as BRD4, TRIM‐24, IKZH1/3 HDAC6, Sirt‐2, and Smad‐3), IV‐metabolic enzymes (DHODH and MetAP2), V‐ Proteins kinases (including, c‐Abl, BKT, ALK, BCR‐Abl CDK9, Akt, and PSD95) and VI‐ proteins targeted neuro‐degenerative illnesses (Huntingtin, a‐synuclein, Tau, and PSD95) as shown in Figure 4.

### 7.1 PROTACs Targeting Estrogen Receptor (ER) for Breast Cancer

Breast cancer, the leading cause of mortality (Khurram et al. 2023), has a common nuclear receptors, such as ER‐α and ER‐β, control the expression of gene ER‐α is the main controller that causes estrogen transduction in the reproductive tract of females and glands in the breasts and plays a role in a variety of biological mechanisms. Over 80% of all breast incidences that have lately been diagnosed are ER‐α positive (ER+) (Pepermans and Prossnitz 2019; Ariazi et al. 2006). Fulvestrant, which selectively degrades the estrogen receptor, is the benchmark treatment for ER+ metastatic breast carcinoma following anti‐estrogen treatment. Although ER has degraded considerably as a result of fulvestrant to achieve the therapeutic intervention, up to 50% of ER may still exist following 6 months of fulvestrant therapy in contrast to baseline values. As a result, even though prescribed treatments have been successful in this clinical setting, some ER+ breast tumors inevitably develop resistance to this form of therapy.

Arvinas has created ARV‐471 (undisclosed structure), a targeted and effective ER degrader for oral delivery to women with metastatic ER+ breast cancer. At 11 nM concentration, ARV‐471 triggered ER destruction in several cell lines of breast cancer. While fulvestrant has a somewhat less effective ability to prevent tumor growth. Additionally, Wang and his colleagues 2019 demonstrated the DC50 levels of ERD‐308, in MCF7 and T47D ER+ breast cancer cell lines, an extremely powerful and efficient ER degrader, respectively (Zhou et al. 2018). ERD308 caused more extensive ER degradation than fulvestrant and had a higher inhibitory effect on cell growth in the cells of MCF7. For the prospective treatment of metastatic and advanced‐stage ER+ breast tumors, these findings introduced a new category of ER degraders.

### 7.2 PROTACs Targeting Androgen Receptor (AR) for Prostate Cancer

The androgen receptor was primarily responsible for prostate cancer. Antagonists that are competitive, like enzalutamide, were the first‐line treatments for prostate cancer by preventing the transcriptional action of the androgen receptor (Sanford 2013; Watson, Arora, and Sawyers 2015). On the other hand, most patients eventually developed resistance against medication following prolonged treatment to antagonists.

Salami and his coworkers carried out a PROTAC (ARCC‐4) in 2018 to target AR derived from enzalutamide (Salami et al. 2018). About 95% of cellular androgen receptors might be destroyed by the low‐nanomolar androgen receptor degrader ARCC‐4. Additionally, ARCC‐4 degraded significant clinical androgen receptor point mutations and had inhibitory effects on the proliferation of prostate carcinoma cells.

Arvinas also created the ARV‐110 (undisclosed structure) PROTAC molecule, which has shown the potential ability in destroying AR and AR mutations when used as an oral treatment. In several cell lines frequently utilized in investigations on prostate cancer, ARV‐110 at 10 nM could destroy 95% to 98% of AR within 24 h (Sun and Rao 2020).

### 7.3 PROTACs Targeting BCR‐ABL for Chronic Myelogenous Lymphoma (CML)

The primary factor for chronic myelogenous lymphoma is the fusion gene BCR‐ABL (CML) (Hantschel and Superti‐Furga 2004). The chromosome 9 ABL gene moves to the chromosome 22 BCR gene, resulting in the development of BCR‐ABL. By stimulating downstream signaling, BCR‐ABL caused CML cells in patients to grow abnormally (Hantschel et al. 2012). ATP‐competitive inhibitors are still the main target for the creation of novel pharmaceuticals to treat CML that target the BCR‐ABL ABL tyrosine kinase. As a result, tyrosine kinase inhibitors of BCR‐ABL were developed and accepted by the FDA for the therapies of CML. In contrast, Patients with specific mutations in the BCR‐ABL tyrosine kinase coding region, during the treatment of kinase inhibitors subsequently developed therapeutic resistance. To combat the rising drug resistance, three generations of Tyrosine Kinase Inhibitor (TKI) inhibitors have been found. Imatinib, a 1st generation TKI (Druker et al. 1996), dasatinib, a 2nd generation TKI (Schittenhelm et al. 2006), nilotinib, a 3rd generation TKI (Weisberg et al. 2006), and ponatinib are a few examples.

### 7.4 Acu

SMARCA 2 and 4 are important therapeutic targets for acute myeloid leukemia (Vangamudi et al. 2015). Melanoma cell lines that are dependent on SMARCA2 and 4, PROTAC generated from SMARCA inhibitors elicited substantial apoptotic death and persistent suppression of cancer cell growth. Multiplex proteomics research revealed that SMARCA2/4 PROTAC could act as a particular degrader in vivo by modestly downregulating cellular proteins beyond the ATPase complex in AML cell lines (Farnaby et al. 2019).

BET (bromodomains and extra terminal) proteins are promising targets for small‐molecule degradation due to their significant roles in triggering carcinogenic transcriptions (Asangani et al. 2014). The BET signaling in AML was found to be reduced by a PROTAC generated from a BET inhibitor, with IC50 values that are lower compared to the equivalent small molecule inhibitor JQ1. In vitro apoptotic death was induced by BET PROTAC in a concentration‐dependent mode. Moreover, it caused tumor regression in mouse xenograft studies without exhibiting any harm (Qin et al. 2018). The effectiveness of the BET PROTAC in the induction of apoptotic death in AML in vitro and preventing cancer development in vivo were to previous findings (Zhou et al. 2018).

AML had already shown pre‐clinical effectiveness when treated with the Wee1 kinase inhibitor AZD1775 (Fu et al. 2018). AML cells were reported to be stopped in the G2/M phase by an AZD1775‐derived PROTAC, which afterward induced apoptotic death, and less significantly, showed off‐target action against ZFP91 (Li et al. 2020; Cancer Genome Atlas Network 2012).

### 7.5 Colorectal Cancer

In CRC, Myc overexpression is frequent (Cancer Genome Atlas Network 2012). BET protein BRD4, an upstream Myc regulator, is a potential therapeutic target (Zuber et al. 2011). In CRC cells and patient‐derived organoids, a BET inhibitor originated from the PROTAC was demonstrated to destroy BRD‐4 and subsequently a dose‐dependent decrease in cellular Myc mRNA and protein levels. Intriguingly, degraded BRD4 was not accompanied by reduced Myc protein in the fibroblasts, demonstrating that in normal tissues, BRD4 is not as important for the Myc transcriptions (Otto et al. 2019). Thus, BET PROTAC demonstrated the ability to target tumors that are highly reliant on BRD4 and showed minimal toxicity in healthy tissues.

Another small‐molecule degradation strategy for CRC was a bioavailable PROTAC produced from deltazinone and directed at the PDE KRAS shuttling component. In CRC cell lines, PDE and PROTAC have lower IC50 values than deltazinone, inhibits RAS signaling, and induction of cell death in vitro dependant on KRAS. Studies on mice with xenografts revealed that PDE‐PROTAC was more effective than deltazinone in preventing tumor growth (Cheng, Li, et al. 2020).

### 7.6 Melanoma

In vitro proliferation of BRAFV600E melanoma cells was recently shown to be inhibited by PROTACs generated from the BRAF inhibitor BI882370. BRAF PROTAC, however, had lower activity because its IC50 was 10 times greater than those of BI882370 (Han et al. 2020). Future study is necessary to determine the mechanisms causing the decreased efficacy. However, it was demonstrated that BRAFV600E degradation causes an immediate decrease in BRAF‐mediated signaling, and the same findings in the cell lines of H29 colorectal cancer were verified that are dependent on BRAF. Other independent studies have reported the in vitro efficacies of BRAF PROTAC, but it has been demonstrated that mutations that the resistance to BRAF PROTAC is conferred by the RAS family proteins, probably via transduction of the CRAF‐mediated alternative signal (Corcoran et al. 2012).

Melanoma cells have high levels of CD147, a member of the superfamily of immunoglobulins and a transmembrane glycoprotein. CD147 is essential for the growth of tumors. Consequently, it represents a possible pharmacological target for melanoma (Muramatsu 2016). According to Chen et al. research's from 2020, pseudoelastic acid B (PAB), a natural chemical, was used to create the first CD147 PROTAC (Zhou et al. 2020). In vitro and in vivo, PROTAC 44 significantly inhibited melanoma cells which also successfully caused CD147 to degrade. The dynamic functions of the CD147 in tumor networking were examined using a gain‐of‐function approach, the scientists believe that a novel anticancer drug could be developed using the PROTAC.

### 7.7 BTK Targeting Lymphoma and Lymphocytic Leukemia

B—Cell Development and immunological responses depend on a non‐receptor tyrosine kinase known as BTK (Bruton tyrosine kinase). The MCL (Mantle cell lymphoma) and CLL (chronic lymphocytic leukemia) have both been treated with the BTK inhibitors, by inhibiting BCR signaling and controlling innate or adaptive immunity (Burger and Wiestner 2018). BTK mutations at the ibrutinib binding region (BTKC481S), however, cause a lot of individuals to develop therapeutic resistance.

To reduce diffuse the cells of the large B cell lymphoma and MCL by eliminating wild type and the C481S mutant BTKs, the scientist created the irreversibly covalently bonded PROTAC such as P‐13I and L‐18I, which are based on the CRBN ligand and an ibrutinib. (Sun, Ding, et al. 2019; Sun et al. 2018). Additionally, PRO TACs (such as the DD04015 and the DD03171) were developed using a new class of noncovalent BTK inhibitors that effectively degrade the BTKs and slow cancer cell development (Huang et al. 2018; Dobrovolsky et al. 2019).

### 7.8 Lung Cancer

KRASG12C inhibitor‐derived PROTAC LC2 degraded KRASG12C in many cell lines of NSCLC to target the prevalent KRASG12C mutation in NSCLC; however, in comparison to the corresponding inhibitor MRTX844, to obtain the same level of Perk inhibition, a medication concentration that was 5 times higher was needed. LC2 significantly decreased KRASG12C's downstream signaling in vitro (Bond et al. 2020). In NSCLC cell lines, geftinib‐derived EGFRL858R‐targeting PROTAC effectively reduced EGFR targeting signaling in a dose‐dependent manner and showed minimal cytotoxicity against wild‐type EGFR (Cheng, Yu, et al. 2020).

It has also been reported that a further derivative of the EGFR allosteric inhibitor JBJ0412502 is the EGFR targeting PROTAC, selectively degrades EGFRL858R/T790M and works in conjunction with osimertinib to reduce the proliferation of cancer cells in Ba/F3 cells (Jang et al. 2020).

### 7.9 PROTACS Targeting EGFR

The EGFR (epidermal growth factor receptor), or an RTK (receptor tyrosine kinase) activates several carcinogenic signals to stimulate cell differentiation and growth. Many epithelial malignancies, including breast and lung cancers, show EGFR gain‐of‐function mutations or overexpression (Samatar and Poulikakos 2014; Ciardiello and Tortora 2008). The use of EGFR inhibitors to treat cancer, such as gefitinib, lapatinib, and afatinib, has been recognized. However, these medications have a poor clinical response due to extreme drug resistance, which could be carried along by medication‐induced EGFR mutations (Chong and Jänne 2013).

PROTACs compound 1/3/4, based on lapatinib, gefitinib, and afatinib, were created by connecting VHL ligands and showed antitumor activity against cell lines of lung and breast tumors (Xie et al. 2023). These PROTACs had a variety of EGFRs as their preferred targets: Compound‐1 killed either exon20 insertion or wild‐type EGFRs, Compound‐2 favored exon19 deletion or the L858R EGFRs, and Compound‐3 destroyed L858R/T790M dual mutant EGFR (Burslem et al. 2018). In lung tumor cell lines with the matching mutation of EGFR, the production of PROTAC‐140 and DDC01163, both demonstrated tumor suppression activity (Jang et al. 2020; Zhang et al. 2020).

### 7.10 PROTACS Targeting BRAF

The RAF family of kinases is essential for controlling the RAS–RAF MEK–ERK pathway, which sends carcinogenic signaling to proliferate cell growth (Samatar and Poulikakos 2014). The RAF gain‐of‐function mutations, such as BRAFV600E, are powerful cancer‐causing agents (Lavoie and Therrien 2015). Although BRAFV600E Inhibitors are extremely effective at treating cancer, their sustainability is constrained by RTKs, RAS activation, or secondary BRAF mutations (Nazarian et al. 2010; Poulikakos et al. 2011). An alternate method to therapeutically restrain oncogenic BRAF is provided by PROTAC (Posternak et al. 2020; Chen et al. 2019). To create the PROTAC P4B, Posterna et al. combined the BRAF inhibitor BI882370 with the CRBN ligand (Posternak et al. 2020). This specifically repressed melanoma and colorectal cancer cells that carried the BRAFV600E or other BRAF mutations.

### 7.11 PROTACs Targeting Cyclin‐Dependent Kinases (CDKs)

In eukaryotes, there is a class of serine–threonine kinases called CDKs. These kinases in mammals could be classified as members of the gene transcription subfamily (CKD7/8/90) based on their biological functions (Li et al. 2016; Whittaker et al. 2017). In light of the critical roles they play in the biological processes, cell cycle, and gene transcription, CDKs have recently been recognized as significant targets for the treatment of cancer. Additionally, various CDK inhibitors, including flavopiridol (Zeidner et al. 2015; Awan et al. 2016), (R)‐roscovitine (Cicenas et al. 2015), palbociclib (de Dueñas et al. 2018), ribociclib, and abemaciclib (Laderian and Fojo 2017), have been proposed as potential antitumor medications.

## 8 Clinical Advancements of PROTAC Therapeutics in Cancers

PROTACs are superior to small‐molecule inhibitors in a number of ways, such as their capacity to target “undruggable” proteins, overcome drug resistance, give extended action, show effectiveness at lower dosages, improve safety, and exhibit improved selectivity (Liu, Zhang, et al. 2022). A heterobifunctionally targeted degrader was first employed in human studies by Arvinas Therapeutics in 2019, and it went into clinical phase I. BCL‐xL, IRAK4, STAT3, BTK, BRD9, MDM2, and other PROTACs have so far advanced to the clinical research and development stage; the majority of these targets are first‐in‐class (Hughes et al. 2021).

The effectiveness, safety, and potential of PROTACs as therapeutic agents were shown in the phase I clinical trials of ARV‐110 and ARV‐471. Metastatic castration‐resistant prostate cancer (mCRPC) is the target of ARV‐110, the first PROTAC created to break down the androgen receptor (AR). The first indication of PROTAC‐mediated protein breakdown in humans was found in phase I studies, where a dose of 420 mg was well tolerated. According to results from phase II trials, 46% of patients with the AR T878X/H875Y mutation had a ≥ 50% decrease in prostate‐specific antigen (PSA) levels (PSA50). Furthermore, two of the seven evaluable patients who met the criteria for RECIST (Response Evaluation Criteria in Solid Tumors) achieved a confirmed partial response (PR), while six of them demonstrated tumor decrease. These results show encouraging first tumor therapy effectiveness (Petrylak et al. 2020).

Research has shifted to investigate PROTACs targeting a broader spectrum of proteins in clinical trials in recent years, motivated by the encouraging clinical results of PROTACs targeting androgen and estrogen receptors. For example, ABT263, a dual inhibitor of BCL‐2 and BCL‐XL, was linked to a VHL ligand to create DT2216, a PROTAC that has progressed to phase I clinical trials for the treatment of small‐cell lung cancer and T‐cell lymphoma (Khan et al. 2019). FHD‐609, which is intended to break down BRD9, is also being researched for its possible use in the treatment of synovial sarcoma. KT‐333, another notable PROTAC that targets STAT3, has been recognized as an orphan medication by the FDA to treat peripheral T‐cell lymphoma (PTCL) (Qi et al. 2021). PROTAC redefines small‐molecule medicines and solves the development challenge of conventionally “undruggable” targets. New PROTACs will continue to be developed for clinical research as a biomedical breakthrough (Békés, Langley, and Crews 2022).

A preclinical study explores the development of novel Son of sevenless homolog 1 (SOS1) degraders using PROTAC technology to target KRAS‐driven cancers. The lead compound, LHF418, demonstrated potent SOS1 degradation with a DC50 of 209.4 nM and a Dmax exceeding 80%. Mechanistic analysis revealed that LHF418 induced a CRBN‐ and proteasome‐dependent degradation of SOS1 by forming a ternary complex with SOS1 and CRBN. Additionally, LHF418 effectively inhibited KRAS‐RAF–ERK signaling and suppressed colony formation in KRAS‐driven cancer cells. These findings position LHF418 as a promising lead compound for developing therapies against KRAS‐driven cancers (Li et al. 2024).

Lu et al. demonstrated the antitumor effects of the BRD4 degrader ARV‐825 in cholangiocarcinoma (CCA). BRD4 expression was elevated in CCA tissues, and ARV‐825 efficiently degraded BRD4, surpassing BRD4 inhibitors OTX‐015 and JQ1. ARV‐825 induced apoptosis, inhibited cell proliferation, and reduced c‐Myc expression, a key BRD4 target involved in cancer progression (Li et al. 2011; Wu et al. 2017). Additionally, ARV‐825 upregulated p21 expression and arrested the cell cycle at the G1 phase. These findings highlight ARV‐825 as a potent therapeutic candidate for CCA through BRD4 degradation and downstream pathway modulation (Lu et al. 2019).

Wang's team developed SHP2‐D26, a PROTAC combining a SHP099‐derived SHP2 inhibitor and a VHL ligand, to target SHP2 for proteasomal degradation. SHP2‐D26 effectively degraded SHP2 protein in esophageal carcinoma (KYSE520) and acute monocytic leukemia (MV4;11) cell lines in a time‐ and dose‐dependent manner. Compared to SHP099, SHP2‐D26 exhibited superior activity in inhibiting ERK phosphorylation and suppressing cell proliferation. These results identify SHP2‐D26 as the first PROTAC degrader of SHP2, highlighting its potential as a therapeutic candidate for both solid and hematological tumors (Wang et al. 2020).

A novel therapeutic nanosystem, Substance P peptide‐polyethylene glycol‐poly(d,l‐lactic acid)‐ARV‐825 (SPP‐ARV‐825), was developed to address the challenges of glioma treatment, including the BBB and tumor‐associated macrophages (TAMs). The system integrates the BRD4‐degrading PROTAC ARV‐825 into a micelle composed of Substance P peptide‐modified polyethylene glycol‐poly (d,l‐lactic acid) (SP‐PEG‐PDLLA) and methoxy polyethylene glycol‐poly(d,l‐lactic acid) (mPEG‐PDLLA), enabling BBB penetration and targeted delivery to brain tumors. SPP‐ARV‐825 effectively reduced tumor cell proliferation, induced apoptosis, and suppressed M2 macrophage polarization by inhibiting interferon regulatory factor 4 (IRF4) transcription and the phosphorylation of signal transducer and activator of transcription 6 (STAT6), STAT3, and protein kinase B (AKT). These findings highlight SPP‐ARV‐825's potential as a promising strategy for glioma therapy (Yang et al. 2022).

An in vivo and in vitro study explores the potential of PROTAC EZH2 degrader‐1 in overcoming chemoresistance in small cell lung cancer (SCLC) with leptomeningeal metastasis (LM). The SCLC cell line H128 was mutated and used to establish a mouse model for LM. Gene expression analysis revealed upregulation of EZH2, SLC44A4, and VEGFA, with SLC44A4 particularly elevated in LM cells. In vitro drug testing showed that combining PROTAC EZH2 degrader‐1 with chemotherapy agents (cisplatin, etoposide, and teniposide) significantly increased drug sensitivity in H128‐LM cells. In vivo experiments with the SCLC‐LM mouse model demonstrated that the PROTAC‐based treatment, combined with chemotherapy, significantly inhibited tumor growth compared to chemotherapy alone (*p* < 0.01). These findings suggest that PROTAC EZH2 degrader‐1 may offer a promising therapeutic strategy for SCLC with LM (Shi et al. 2024).

## 9 Advantages of PROTACS

Over the past two decades, PROTAC technology has achieved remarkable milestones, revolutionizing the field of drug discovery. Notably, the first and second oral PROTACs have advanced into clinical trials, showcasing its potential. However, like any innovative technology, PROTAC faces unique challenges and opportunities.

### 9.1 Low Immunogenicity and Minimal Transcriptome/Genome Impact

PROTACs use regulatory ligands for E3 ubiquitin ligases and POIs connected by a linker, enabling precise regulation of POI‐related cellular functions and intracellular biological processes. These chimeric molecules are low‐immunogenic and deplete target proteins having minimal effects on the genome and transcriptome, such that this technique is appropriately applied in vivo where it stands out compared with nucleic acid‐based approaches that include CRISPR‐Cas9 or RNA interference (Moreau and Coen 2020; Xiong et al. 2022).

### 9.2 Recyclability and Catalytic Protein Degradation

PROTACs are reusable after POIs ubiquitination and degradation; thus, PROTACs can perform catalytic exclusion of numerous target proteins, unlike irreversible small‐molecule inhibitors that are not substrates for reuse. PROTACs result in the loss of POI function through the chimera/E3 ligase/POI ternary complex which is reversible and fast.

### 9.3 Overcoming Resistance via Protein Degradation

Since their mechanism involves transient binding, PROTACs are less prone to develop resistance through mutations than small‐molecule inhibitors that require a longer occupation time (Moreau and Coen 2020; Burslem and Crews 2020). PROTACs can be employed to proactively target resistance mechanisms by degrading proteins that drive therapy resistance. For example, a PROTAC targeting BRD4 restored doxorubicin sensitivity in resistant cancer cells and is of potential use in combination therapies (Wagle et al. 2011; Weinstein 2002). MUC1‐C targeting prevented BRAFV600E inhibitor resistance in metastatic colorectal cancer and restored sensitivity in resistant cells (Ciombor and Strickler 2022; Schirripa et al. 2019; Morimoto et al. 2023).

### 9.4 Enhanced Selectivity and Specificity

Proximity between PROTACs, POIs, and E3 ligases contributes to ternary complex stability possibly enhancing selectivity than conventional inhibitors where POIs share conserved active sites (Wang et al. 2021; Koroleva et al. 2022). Structurally optimized PROTACs do not have off‐target activity, and therefore, are less toxic to normal proteins. This specificity enables use of effective concentrations that are now significantly lower than those used in typical inhibitors and thus reducing side effects. For example, BRAFV600E‐targeted PROTACs removed mutant proteins without affecting the normal cells (Alabi et al. 2021; Posternak, Tang, and Maisonneuve 2020).

### 9.5 Targeting “Undruggable” Proteins

**FIGURE 5:**
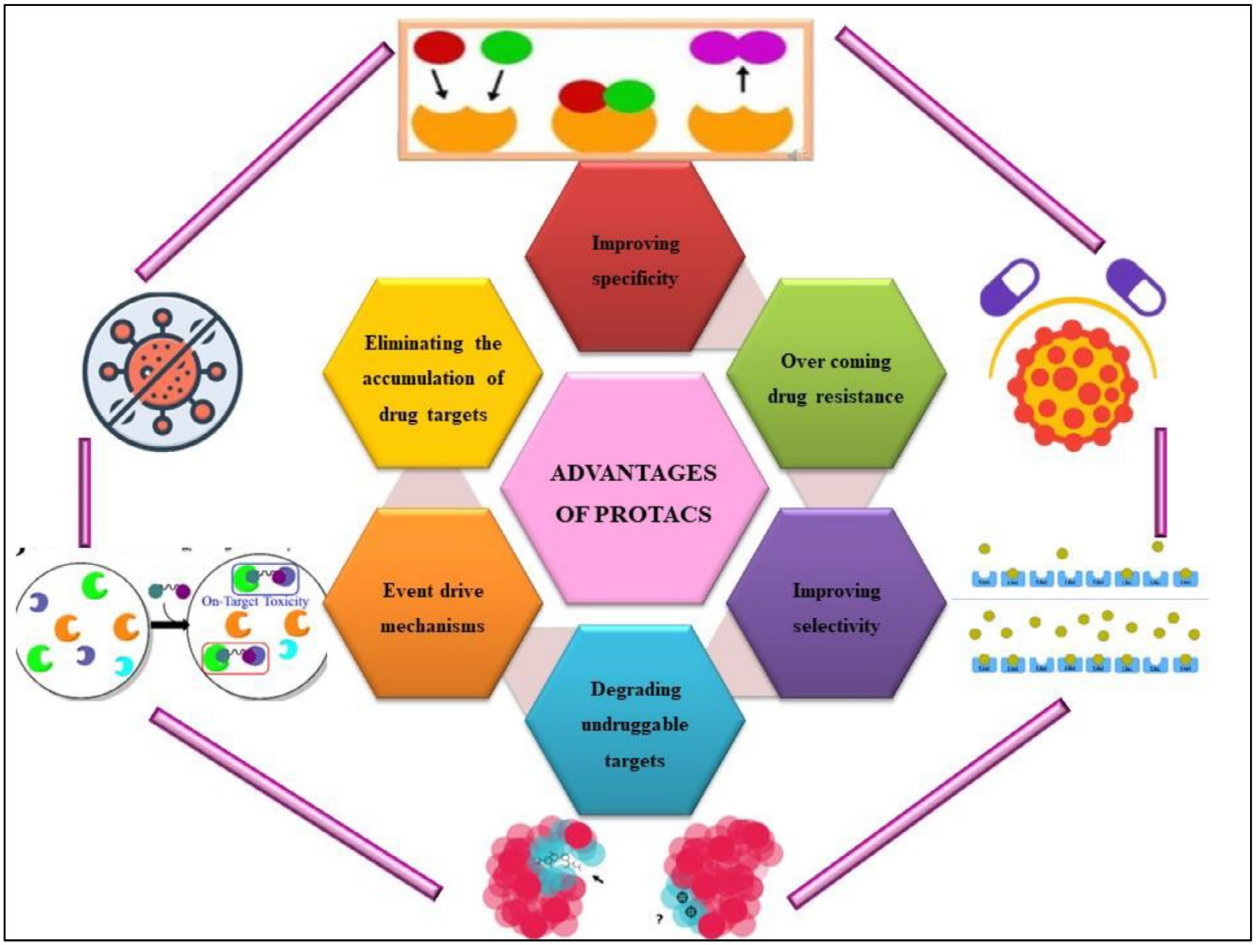
Different advantages of PROTACS.

PROTACs can facilitate the degradation of target proteins, including those previously deemed “undruggable” as shown in Figure 5. This innovative approach offers a new strategy for developing novel small‐molecule drugs, addressing the challenges associated with traditional targeted therapies (Röth and Fulcher 2019). PROTACs provide a therapeutic option for previously undruggable oncoproteins like MYC and STAT3. They offer increased target specificity and the ability to degrade mutant proteins in resistant cells. For instance, ARV‐110 reduced androgen receptor abundance in resistant prostate cancer models (Gao et al. 2022). Similarly, the KRASG12C‐specific PROTAC, LC‐2, demonstrated mutant‐specific degradation without affecting wild‐type KRAS, even in resistant cell lines (Bond et al. 2020; Hallin et al. 2020).

### 9.6 Dual and Multitarget PROTACs

Dual PROTACs, for example, PROTAC 753B, degrades two targets at once: BCL‐XL and BCL‐2 while reducing the unwanted side effect of thrombocytopenia using tissue‐specific E3 ligases (Jia et al. 2023). Multitarget PROTACs, like Y‐PROTAC, selectively activate in glutathione‐rich tumors while reducing off‐target effects (Wang et al. 2023). These approaches improve the therapeutic efficacy for cancers that are characterized by high intratumoral heterogeneity, such as chromosome instability (CIN)‐positive cancers (McClelland 2017).

### 9.7 Avoiding Compensatory Protein Expression

Targeted therapies, including small‐molecule inhibitors, can stimulate compensatory protein expression, decreasing efficacy and increasing toxicity (Lu et al. 2015). For example, statin therapy leads to increased HMG‐CoA reductase (HMGCR) expression by upregulating transcription and slowing its degradation, thereby limiting the effectiveness of statins for cardiovascular disease treatment (Lu et al. 2015). PROTACs overcome compensatory protein expression through the enhancement of UPS‐dependent degradation and offer an acute, reversible means to deplete proteins, making them valuable tools for understanding protein function (Burslem and Crews 2020).

### 9.8 Targeting Cancer Cell Proliferation and Apoptosis

Addressing hyperactivated growth signals that promote unchecked tumor cell development, like the RAS–RAF–MEK–ERK pathway, is necessary to target cancer cell proliferation (Martin 2003; Samatar and Poulikakos 2014). Proteins involved in controlling the cell cycle that are overexpressed, overactivated, or mutant have been broken down using PROTAC technology. Cancer cells avoid death by either downregulating pro‐apoptotic factors (like Puma, Bax) or upregulating anti‐apoptotic proteins (like Bcl‐2, Bcl‐xL), which leads to aberrant survival, resistance to treatment, and recurrence (Carneiro, El‐Deiry, and Oncology 2020; Mohammad et al. 2015). Apoptosis can be targeted to induce death of cancer cells and improve their response to anticancer treatments.

### 9.9 Targeting Cancer Angiogenesis

Angiogenesis targeting is critical since tumors depend on neovasculature, produced by angiogenesis, for nutrient and oxygen delivery while removing waste (Hanahan and Weinberg 2011). The process is initiated by hypoxia through the induction of growth factors, such as vascular endothelial growth factor (VEGF), which activates blood vessel formation by binding to VEGF receptors, VEGFR (Apte, Chen, and Ferrara 2019). Inhibiting the VEGF/VEGFR pathway has formed the cornerstone of anticancer therapy. VEGFR‐2 is the most promising target because it primarily promotes endothelial cell proliferation and angiogenesis. Shan et al. developed PROTAC‐2 and PROTAC‐5 based on S7, a known inhibitor of VEGFR‐2. These demonstrated excellent VEGFR‐2 degradation and anti‐proliferative activity in endothelial cells with very minimal toxicity to VEGFR‐2‐negative HEK‐293 cells, ensuring excellent safety (Shan et al. 2020).

### 9.10 Targeting Cancer Metastasis

Metastasis accounts for ~90% of cancer‐related deaths globally. It is associated with tumor cell dissemination, extravasation, and colonization of distant organs via circulatory systems (Vanharanta and Massagué 2013; Reymond, d'Água, and Ridley 2013). An important process during metastasis is epithelial‐to‐mesenchymal transition (EMT), which is induced by signaling pathways like the Integrin/FAK/PI3K/AKT axis (Hamidi and Ivaska 2018; Singh et al. 2018). In the past decade, PROTACs against EMT‐related proteins have emerged as promising strategies for the control of metastasis.

### 9.11 Future Challenges of PROTACS

Most PROTAC molecules exist today outside of the traditional Lipinski “rule‐of‐five” space (Edmondson et al. 2019). As a result, PROTAC faces particular difficulties in their development as possible medicinal agents. To clarify the absorption, distribution, metabolic reactions, elimination, and cytotoxicity of PROTAC, further research and study would be needed because the process by which it breached the cell membrane is yet not sufficiently understood. Finding molecules with optimal physicochemical qualities and boosting PROTAC bioavailability and cell absorption to sustain the level required for pharmacological efficacy remain two of the most difficult challenges. Compound RC32's capacity to quickly and irreversibly cause the breakdown of the target protein FKBP12 in living organisms demonstrated that the permeability of cells of the PROTAC can be enhanced by employing the appropriate linker (Edmondson et al. 2019). The very first oral androgen receptor PROTAC degrader treating prostate tumors has recently made clinical progress, demonstrating that pharmacokinetic challenges such as limited bioavailability and poor permeability may be addressed. Smaller POI ligands, like non‐functional ligand precursors, can be chosen in parallel to strike an equilibrium between affinity and ligand optimization. By preserving adequate target affinity while lowering molecular weight, these ligands may be better candidates for PROTAC development, enhancing pharmacokinetic characteristics in the process (Paiva and Crews 2019).

Medicinal chemists face a significant problem due to the intricacy of PROTAC molecules. conjugation vector structure, Linkage site, and connector length all play a significant role in the production of productive PROTAC but have proven to be difficult to address systematically (Jiang et al. 2019). The length and nature of the linker determine a PROTAC's rigidity, hydrophobicity, and selectivity. Optimization of the linker is often done by making multiple PROTACs and varying the linker parameters for structure–activity relationship studies that are both time‐consuming and expensive. Recently, a multidisciplinary approach based on structural biology and computation has been reported by Bemis et al., which simplifies this approach. Although this method does not completely eliminate the empirical nature of SAR studies, it is a very promising approach to reduce the number of molecules needed and speed up the determination of the optimal linker length and properties (Bemis, La Clair, and Burkart 2021).

To activate the intracellular UPS, PROTAC must penetrate cells, the permeability of its membrane is essential to its operation. The PROTAC penetration mechanism is still unknown at this time. Since the majority of recognized PROTACs have molecular weights between 1000 and 2000 Da (Cecchini et al. 2021; Powell et al. 2018), passive diffusion and active transport are the primary methods by which they enter cell membranes. Nevertheless, PROTACs have worse cell/tissue permeability than tiny molecules due to their amount of exposed polar surface and the large molecular weight. The permeability of PROTACs has been increased using a variety of methods. Common techniques include keeping the molecule's mass below 1000 Da, (Klein et al. 2020) and resulting in the production of mature PROTAC in cells by cleaving it into 2 small precursors (Lebraud et al. 2016). Furthermore, the addition of extended flexible linkers to create hydrogen bonding which partly lessens polarities may increase the PROTAC's permeability. (Liu, Chen, Kaniskan, et al. 2021), likewise might be achieved by combining E3 ligands with a cell‐permeable peptide (Schneekloth Jr et al. 2004). In addition to altering PROTAC itself, PROTAC absorption by cells was greatly boosted when transported by liposomal nanoparticles (Liu, Chen, Kaniskan, et al. 2021).

The development of PROTACs is now heavily dependent on the discovery and optimization of known POI/ E3 ligands because they are used as protein decoys in the PROTACs design. Furthermore, some POI/E3 ligands are known to have poor specificity, which causes such PROTACs to have off‐target impacts (Lai, Crews, and Discovery 2017). Finding extremely precise POI/E3 ligands is therefore crucial for developing potent drugs. New E3 ligases can be identified and used to degrade more proteins while potentially allowing tissue‐ or organ‐specific degradation, hence minimizing systemic effects by the differential expression of E3 ligases. Optimal scaffolds to recruit CRBN E3 ligase have also been reported in patents, and the phthalimide moiety has been replaced by better pharmacokinetics and stability. Selectivity is still vital, as PROTACs should aim at specific tissues to limit the side effects. Crew et al. (Békés, Langley, and Crews 2022) summarize the future of targeted protein degradation (TPD) focusing on the demand for the discovery of tissue‐selective E3 ligases as well as widening applications beyond oncology.

In order to optimize PROTAC development, generate useful ternary complexes, and forecast effective protein degradation results, structure‐based drug design provides *in silico* methods. These techniques help choose lysine residues, spacer anchor points, and POI binding pockets for ubiquitin transfer, tackling issues such as dual Bcl‐xL/Bcl‐2 degraders. In order to streamline rational PROTAC design, the PROTAC‐Model protocol has recently integrated tools such as FRODOCK, RDKit, VoroMQA, and RosettaDock to effectively model near‐native ternary complex structures (Lv et al. 2021; Weng et al. 2021; Ramírez‐Aportela, López‐Blanco, and Chacón 2016; Olechnovič and Venclovas 2017; Lyskov and Gray 2008). Exploration of PROTAC chemical space involves using physicochemical descriptors to understand the role that the linker plays in modifying properties. The PROTACpedia database helps in linking those descriptors to degradation activity; Ermondi et al. used the PROTAC‐DB database in elucidating 2D structures of ~1600 PROTACs, 60 ligase ligands, 800 linkers, and 202 POI ligands (Guedeney et al. 2023; Ermondi and Garcia‐Jimenez 2021). Ishida et al. have highlighted approaches including DNA‐encoded libraries, display technologies, cyclic peptides, molecular glue degraders, and covalent warhead ligands for the expansion of PROTAC chemical space (Ishida and Ciulli 2021).

## 10 Conclusion

In summary, PROTACs have emerged as a revolutionary approach in cancer therapy, selectively degrading oncogenic proteins with diverse targets. Their unique advantages, including lower concentrations, reduced cytotoxicity, and the ability to tackle traditionally challenging targets, make them promising candidates for personalized cancer treatment. Ongoing studies reveal encouraging results across various cancers, signaling a potential paradigm shift. Despite challenges in optimization, the development of oral androgen receptor PROTACs is a notable achievement. Continued research is crucial to unlock the full therapeutic potential of PROTACs, shaping the future of targeted protein degradation in cancer therapy.

## Author Contributions

**Saooda Ibrahim:** conceptualization (equal), writing – original draft (equal), writing – review and editing (equal). **Muhammad Umer Khan:** supervision (equal). **Iqra Khurram:** investigation (equal). **Raima Rehman:** investigation (equal). **Abdur Rauf:** investigation (equal). **Zubair Ahmad:** investigation (equal). **Abdullah S. M. Aljohani:** investigation (equal). **Waleed Al Abdulmonem:** investigation (equal). **Mohammed Mansour Quradha:** investigation (equal).

## Ethics Statement

No animal and human participants were involved in this study so ethical approval was not applicable in this research project.

## Conflicts of Interest

The authors declare no conflicts of interest.

## Data Availability

No data associated in the manuscript.
